# Supplementary material for: Informed Consent for Newborn Genomic Screening: Interest-Holder Perspectives on Dynamic Consent in an Evolving Landscape
Source: Int J Neonatal Screen. 2025 May 28;11(2):41. doi: 10.3390/ijns11020041 (PMC12193422; doi:10.3390/ijns11020041)
Supplement: Supplementary file 1 [file IJNS-11-00041-s001.zip › Interview Guide.pdf]

**Table S1**

*Interview Guide*

| **Interview questions** |
| --- |
| What do you consider to be your role in newborn bloodspot screening or genomics? |
| Are you familiar with the current consent process for NBS? |
| - If YES, how do these guidelines inform your work? |
| What are your views on the current consent information and procedures for NBS? |
| In your work, what kinds of conversations about consent come up? |
| - What is your experience when you have discussions about consent? |
| It appears we will be moving more towards genomic NBS, what might this mean for your work? |
| - To improve consent processes what do you think would be the most important factors to consider? |
| - Would this be a difficult or easy procedure to explain? |
| Reflecting on your experience, what are your thoughts about having different consent processes between states and territories? |
| - What is your view on having a nation-wide consent process? Would it be feasible? |
| - What would be the benefits or challenges of making consent processes the same nation-wide? |
| **Introduction of CoGenT** (after checking if participants watched the video) |
| How would you feel if a dynamic consent platform like CoGenT was used in the newborn context to facilitate informed decision making? |
| What do you think about online consent platforms – in terms of their potential role in helping parents consent to genomic screening? |

What do you perceive to be any positive or negative consequences of using a platform like CoGenT?

- How do you think parents may respond to the use of CoGenT?
- While CoGenT seeks to improve the consent experience, considering the fact the current screening rate is over 99% nation-wide, how might an introduction of dynamic consent impact screening rates?

When might be the most appropriate time to introduce CoGenT and information about NBS to parents and why?

What might need to be considered when adapting a platform like CoGenT to Indigenous populations or CALD populations?

To what extent do you feel key stakeholders like yourself would get onboard with the implementation of CoGenT, keeping in mind it has been developed for oncology?

What kind of infrastructure or system changes would be needed to accommodate the implementation of a platform like CoGenT?

To what extent do you think CoGenT could be used to streamline consent processes nation-wide? How?

---
